# Impact of Treating Asymptomatic Bacteriuria in Kidney Transplant Recipients: A Prospective Cohort Study

**DOI:** 10.3390/antibiotics10020218

**Published:** 2021-02-22

**Authors:** Sara Fontserè, Carmen Infante-Domínguez, Alejandro Suárez-Benjumea, Marta Suñer-Poblet, Carmen González-Corvillo, Guillermo Martín-Gutiérrez, Gabriel Bernal, Jerónimo Pachón, María Eugenia Pachón-Ibáñez, Elisa Cordero

**Affiliations:** 1 Unit of Infectious Diseases, Microbiology, and Preventive Medicine, Virgen del Rocío University Hospital of Seville, 41013 Seville, Spain; sarafontsere@gmail.com (S.F.); carmeninfanted@gmail.com (C.I.-D.); guiller_mg86@hotmail.es (G.M.-G.); gabrielbernalblanco@gmail.com (G.B.); pachon@us.es (J.P.); elisacorderom@gmail.com (E.C.); 2 Urology and Nephrology Unit, Virgen del Rocío University Hospital, 41013 Seville, Spain; alejandro.suarez.sspa@juntadeandalucia.es (A.S.-B.); msuner@us.es (M.S.-P.); carmen.gonzalez.corvillo.sspa@juntadeandalucia.es (C.G.-C.); 3 Department of Medicine, University of Seville, 41009 Seville, Spain; 4 Institute of Biomedicine of Seville (IBiS), Virgen del Rocío University Hospital/CSIC/University of Seville, 41013 Seville, Spain

**Keywords:** urinary tract infections, kidney recipients, asymptomatic bacteriuria, cystitis, prospective observational cohort

## Abstract

This study aims to define the epidemiologic, clinical, and microbiological features of asymptomatic bacteriuria (AB) and cystitis in kidney transplantation recipients (KTRs), and to determine the impact of antimicrobial therapy of AB and the risk factors of cystitis. We conducted a prospective observational study of AB and cystitis in KTRs from January to June 2017. One-hundred ninety seven KTRs were included: 175 (88.8%) with AB and 22 (11.2%) with cystitis. The most frequent etiologies were *Escherichia coli*, *Klebsiella* *pneumoniae*, *Enterococcus* *faecalis*, and *Pseudomonas aeruginosa*. No differences were observed regarding the etiologies, antimicrobial susceptibility patterns, and microbiologic outcomes in AB vs. cystitis. The treatment of AB diminished the microbiological cure and increased the rates of microbiologic relapses and reinfections; in addition, treated AB patients showed a trend of developing symptomatic urinary tract infection in the following six months. The analysis of the data identified the following independent risk factors for cystitis during the six months of follow-up: AB treatment, thymoglobulin induction, previous acute pyelonephritis, and time since transplantation < 1 year. In summary, considering the lack of clinical benefits of treating AB and its impact on cystitis development in the follow-up, we support the recommendation of not screening for or treating AB.

## 1. Introduction

Despite improved surgical techniques, antimicrobial prophylaxis, new immunosuppressive therapies, and better hygiene management of solid organ transplantation recipients (SOTRs), infectious complications remain a major cause of morbimortality in these patients. Urinary tract infections (UTIs) are among the most common infectious complications and the first cause of antibiotics treatment in kidney transplantation recipients (KTRs). The reported incidence of UTIs ranges from 4% to 75% in kidney recipients; this wide range could be explained by the heterogeneity of definitions of UTIs, follow-up times, surgical techniques, antimicrobial prophylaxis and immunosuppressive drugs, and design of the studies [1,2,3,4,5].

The real effect of UTIs on the outcome of KTRs is not clear. Some studies suggest the absence of association of asymptomatic bacteriuria (AB) and acute cystitis with allograft survival, rejection, renal function, and all-cause short-term mortality. The screening and treatment of AB did not improve the early outcome after transplantation and increased the risk of suffering multidrug-resistant (MDR) infections in several studies [6,7]. 

On the other hand, other studies have reported that the burden of UTIs in KTRs is real and high, as suffering from a UTI during the first year after transplantation increases mortality (41%) and costs per event [8]. Moreover, these patients have a higher risk of MDR infections, which also compromise the outcome [6]. The impact of acute pyelonephritis (APN) on allograft function, although uncertain, determines an adverse outcome when occurring early after transplantation [4,5,9,10].

Despite their clinical frequency, there are unanswered key points regarding the epidemiology of UTIs in KTRs, the differences in resistance patterns depending on the presence of symptoms, and the impact of treatment in the allograft survival, rejection, and mortality. In this study, we aimed to evaluate the epidemiology and clinical manifestation of UTIs and the impact of antimicrobial therapy on AB in kidney transplantation recipients.

## 2. Methods

### 2.1. Study Design

A prospective observational cohort of consecutive cases of all uncomplicated-cystitis and AB cases in KTRs that attended the outpatient clinic from January 2017 to June 2017, at the Virgen del Rocío University Hospital, Seville, Spain, was analyzed. For the study, only the first episode, in patients with reinfections or relapses, was analyzed. All interventions followed standard clinical practice. The decision to treat the AB and cystitis episodes and the antimicrobial therapy was the choice of the physicians in charge of the patients.

Urine samples were processed within 4–8 h after collection and urine pH was measured. The Microbiology Service identified the bacterial isolates and performed susceptibility testing by conventional biochemical tests (biochemical testing, pigment production, growth, and colony characteristics). The causative organism and antibiogram were identified using the MicroScan WalkAway^®^ plus System (Beckman Coulter, Switzerland). When the identification was uncertain, it was confirmed by the Bruker Biotyper MALDI-TOF MS system (Bruker Daltonik GmbH, Leipzig, Germany). The European Committee on Antimicrobial Susceptibility Testing (EUCAST) criteria for categorizing susceptibility and resistance patterns were used [11]. Plasma creatinine, and urinary pH, leukocyturia, and nitrites determinations were also performed at inclusion. Demographics, chronic underlying diseases, time from the kidney transplantation, immunosuppressive regimens, clinical data, and antimicrobial therapy were recorded in a standardized database.

Patients were follow-up for six months after inclusion. Urine cultures were performed one and six months after inclusion. Moreover, in patients with urinary symptoms during the follow-up, urine cultures were also performed.

The study was approved by the Ethics Committee of the University Hospitals Virgen del Rocío and Virgen Macarena (2016/186), Seville, Spain.

### 2.2. Definitions

GESITRA/REIPI UTI guidelines [12] were used: Bacteriuria: Urine specimens isolated in quantitative counts ≥ 10^5^ colony-forming units (CFU)/mL. Asymptomatic bacteriuria: The presence of bacteriuria in the absence of any symptoms of a UTI. Cystitis: Bacteriuria and clinical manifestations such as dysuria, pollakiuria, urinary urgency, suprapubic pain, and/or hematuria, in the absence of pyelonephritis symptoms. *Acute pyelonephritis:* The simultaneous presence of bacteriuria and/or bacteremia and fever, with one or more of the following: Lumbar pain (if native kidney involved), renal allograft tenderness (if transplanted kidney involved), chills, or cystitis symptoms. *Clinical cure*: The resolution of symptoms at seven days after inclusion. *Microbiological cure (eradication)*: Negative urine culture at 7–9 days after the end of treatment. *Microbiological reinfection*: New episode of infection by a different pathogen than the initially isolated. *Microbiological relapse*: Detection of the same initial pathogen during the month after the inclusion, with a sterile culture before. *Microbiological persistence*: No negative urine culture in the follow-up. *Mortality*: Death during the six months prior to the follow-up. *Impairment of renal function*: Elevation of creatinemia ≥ 0.5 mg/dl.

### 2.3. Statistical Analysis

A descriptive statistical analysis was performed. Continuous variables were expressed as median and interquartile range or mean and standard deviation if adjusted to a normal distribution and evaluated by Shapiro–Wilk or Kolmogorov–Smirnov tests when appropriate. For bivariate analysis, the chi-square test or the Fisher exact test was used for categorical variables; Bonferroni correction was applied when appropriate. For quantitative variables, the Mann–Whitney test or Student’s t-test were used based on their distribution. If the variance was not homogeneous (Levene test), an ANOVA test was applied. The relative risks were expressed as odds ratios (ORs) and 95% confidence intervals (CI). Multivariate models were used to adjust for possible confounding variables. The clinically relevant and statistically significant variables found in the bivariate analysis were included in a matrix analysis (checked by chi-square test for categorical variables and Student’s t-test for quantitative variables). Only the independent variables were finally included, which was the multivariate model that described the outcome better. Significance was established at *p* < 0.05. All reported *p*-values are based on two-tailed tests. Statistical analyses were performed using SPSS version 18.0 software (SPSS, Chicago, IL, USA).

## 3. Results

### 3.1. Characteristics and Outcomes of the Entire Cohort

Our study included a total of 197 patients, 175 (88.8%) with AB and 22 (11.2%) with cystitis. Out of the total, 104 (52.8%) were women, and the median age was 59 years (IQR: 48–69). The median time after transplantation was 3.8 years (IQR: 0.8–10). Our study included 58 (29.4%) and 10 (6.9%) patients who received the transplantation in the previous year and month, respectively. The most common immunosuppressive drug combination was mycophenolate (MMF), prednisone, and tacrolimus (*n* = 124, 62.9%). Induction therapy was performed for 91 (46.1%) patients: 67 (34.0%) with basiliximab or daclizumab and 24 (12.1%) with thymoglobulin. Instrumentation of the urinary tract took place in 40 (20.3%) patients, 24 (12.2%) within the previous six months of the UTI diagnosis.

In the previous six months, 80 patients were diagnosed with at least one episode of bacteriuria (40.6%), 15 with APN (7.6%), and 10 with cystitis (5.1%). Patients with cystitis had more frequently detectable urinary nitrites than patients with AB (35.7% vs. 9.1%, *p* = 0.01, OR 3.4, 95% CI 1.3–9.1). At inclusion, 70 (35.5%) patients had viral co-infections: Cytomegalovirus infection in 45 (22.8%) cases and BK virus infection in 19 (9.6%). Patients with cystitis were more frequently co-infected with the hepatitis C virus (HCV) (33.3% vs. 3.3%, *p* = 0.01, OR 2.3, 95% CI 2.3–18.5). No differences were found in other demographics and transplant-related variables (Table 1).

Most frequent etiologies in all patients were *Escherichia coli* (*n* = 89, 45.2%), *Klebsiella pneumoniae* (*n* = 30, 15.2%), *Enterococcus faecalis* (*n* = 23, 11.6%), and *Pseudomonas aeruginosa* (*n* = 13, 6.6%) (Table 1). Etiologies were similar in patients with cystitis and AB. There were 60 (30.4%) isolates resistant to cotrimoxazole, 55 (27.9%) to ciprofloxacin, 38 (19.2%) to amoxicillin-clavulanate, 21 (10.6%) to third- and/or fourth generation cephalosporins, and 19 (9.6%) to fosfomycin. No differences were observed in antimicrobial susceptibility between isolates from patients with cystitis and AB (Table 1).

Seventy-five (38.1%) patients received antimicrobial treatment, with differences in AB (*n* = 54, 30.8%) and cystitis (*n* = 21, 95.4%) cases. The most common antibiotics prescribed were fosfomycin, ciprofloxacin, and amoxicillin-clavulanate, without differences between cystitis and AB (Table 1).

At the one-month follow-up, 191 (96.9%) out of the 197 patients were cured, and 4 (2.0%) and 2 (1.0%) had cystitis and APN, respectively, without differences between patients with AB or cystitis at inclusion. At the six-month follow-up, 181 (91.8%) patients were cured, and 8 (4.0%) and 8 (4.0%) had cystitis and APN, respectively, without differences between patients with AB or cystitis at inclusion (Table 2). The most frequent etiologies of symptomatic UTIs during the follow-up were *E. coli* (64.7%) and *E. faecalis* (29.2%).

Regarding the microbiological outcome, at the one-month follow-up, 111 (56.3%) patients were microbiologically cured. In 40 (20.3%) patients, the bacteriuria persisted, 11 (5.5%) patients relapsed, and 14 (7.1%) patients were re-infected; 21 (10.6%) patients had no urine cultures at this time. At the six-month follow-up, 53 (26.9%) were cured, and 34 (17.2%), 27 (13.7%), and 37 (18.7%) had persistence, relapse, and re-infection, respectively. No differences were found in microbiological cure at any follow-up time-points regarding AB or cystitis diagnosis at inclusion (Table 2).

At the six-month follow-up, renal function worsened in 10 (5.1%) patients, four (2.0%) had a graft rejection, and one (0.5%) lost the graft. Six (3.0%) patients died during the six months of follow-up (five AB and one cystitis), none because of the UTI. The graft and survival outcomes of patients with AB and cystitis were similar (Table 2).

### 3.2. Impact of Antibiotic Treatment in AB Outcome

Among patients with AB, 54 (30.8%) received antimicrobial therapy; most common treatments were fosfomycin (*n* = 19, 10.8%), ciprofloxacin (*n* = 16, 9.1%), and amoxicillin-clavulanate (*n* = 12, 6.8%). A higher proportion of treated AB patients, when compared to those untreated, received the transplant in the six months before inclusion (30.2% vs. 16.7%, *p* = 0.04, OR 2.16, 95% CI 1–4.6) and had isolates resistant to cotrimoxazole (54.1% vs. 23.2%, *p* < 0.01, OR 2.24, 95% CI 1.4–3.4). Moreover, there were trends to higher creatinine levels before and during the actual episode in vs. untreated AB patients: 1.78 vs. 1.54 mg/dL *(p* = 0.07) and 1.77 vs. 1.57 mg/dL (*p* = 0.08), respectively. No differences were found among the rest of the analyzed variables (Table 3).

The episodes of symptomatic UTIs in AB patients during the follow-up are summarized in Table 3. After one month, 4 (7.4%) out of 54 treated AB patients presented with a symptomatic UTI (two cystitis and two APN), whereas 1 (0.8%) out of 121 untreated AB patients had a cystitis episode (*p* = 0.06). After six months, 6 (11.1%) treated vs. 4 (3.3%) untreated AB patients had UTI episodes (*p* = 0.07, OR 3.65, 95% CI 0.98–13.53).

A multivariate analysis was performed to evaluate possible confounding variables of the effect of treating AB at the risk of developing symptomatic UTIs in the six months after inclusion. The following variables were identified as independent risk factors: Use of thymoglobulin as the induction drug (*p* < 0.01, OR 8, 95% CI 1.9–34.2), APN after the transplant (*p* < 0.01, OR 12, 95% CI 2.7–53.5), antimicrobial treatment of AB (*p* = 0.02, OR 5, 95% CI 1.2–20.6), and time since transplantation less than one year (*p* = 0.01, OR 5.7, 95% CI 1.5–22.2) (Table 4).

Regarding the microbiological outcome, at one-month follow-up, patients with treated AB experienced a microbiological cure less frequently than those untreated (44.4% vs. 61.9%, *p* < 0.01, OR 0.49, 95% CI 0.25–0.94). These patients also had a higher number of relapses (12.9% vs. 3.3%, *p* < 0.05, OR 2.2, 95% CI 1.3–3.7), and re-infections (12.9% vs. 2.4%, *p* < 0.01, OR 2.4, 95% CI 1.5–3.9). At the six-month follow-up, microbiological outcomes were similar in treated and untreated AB (Table 3).

## 4. Discussion

This study shows that antimicrobial resistance is a major issue in kidney recipients with a UTI and that treating AB in kidney recipients diminish the microbiological cure and increases the rates of microbiologic relapses and reinfections; in addition, treated AB patients showed a trend of developing symptomatic UTIs in the following six months. To our knowledge, this is the largest study to examine the epidemiology and clinical manifestation and impact of antimicrobial therapy on non-complicated UTI in kidney recipients, prospectively.

The most common etiology of non-complicated UTI was *E. coli*, as described for the general population [13,14]; however, the spectrum of etiologies was more diverse than in non-immunocompromised hosts, with a higher frequency of *Enterococcus* spp. and *Pseudomonas aeruginosa* infections [1,15]. The high proportion of antimicrobial resistance found must also be highlighted; it is in the range of the proportion described in other studies of kidney recipients (44–77%) [15,16,17] and clearly higher than the incidence described in the general population (18–25%) [14,18].

One-third of the episodes included occurred during the first year after the transplant. It has been described that most episodes of bacteriuria occur early after transplantation [13]. Several reasons have been hypothesized to explain these findings, including immunological net status or urinary instrumentation. The close follow-up of early kidney recipients could also have contributed to this finding.

No differences were found regarding the etiology and antimicrobial susceptibility of bacteriuria according to the presence of symptoms; however, in patients with bacteriuria, the presence of nitrites was associated with urinary symptoms. This finding has not been previously described in kidney recipients; however, a higher sensitivity of urinary nitrites at diagnosing cystitis when screening for bacteriuria in pregnant women, rather than AB, has been reported [19].

Hepatitis C virus co-infection also occurred more frequently in patients with cystitis. In the RESITRA cohort, an association is reported between hepatitis C virus serostatus and receiving thymoglobulin or experiencing an upper UTI, which are, at the same time, risk factors for developing a symptomatic UTI in our cohort [20].

Treating AB did not improve 1-month and 6-month microbiological outcomes. It did not have any impact on the survival of either patients or grafts. This is in accordance with what has been previously described: The persistence of bacteriuria, relapse, or reinfection did not affect the survival, renal function, or allograft function [2,4,10,21,22,23]. Preventive measures to reduce UTIs in transplant recipients, as antimicrobial prophylaxis, have not been reported to affect either the graft’s or patient’s survival; however, antimicrobial prophylaxis reduced the incidence of bacteriuria and sepsis in a meta-analysis study [24].

In the present study, treating AB did not prevent the development of symptomatic UTI in the follow-up, as other studies had already reported [4,25,26]. On the contrary, independently treating AB increased the risk of symptomatic UTIs in the following six months. Some factors were also identified to independently increase the risk of developing a symptomatic UTI during the six months of follow-up: Induction therapy with thymoglobulin, “early” post-transplant period bacteriuria, and previous APN. These factors might be, as previously stated, surrogate markers of the global net-state of immunosuppression and urinary predisposing factors, which might have contributed to a higher risk of symptomatic infection [2,3,8].

The open design of the study is a limitation to be considered. It might explain the increased risk of symptomatic UTI in the follow-up in treated patients with AB. The physician in charge of the patient decided when to treat and, therefore, they more frequently treated patients who had AB, were recently transplanted, had previous episodes of APN, or received thymoglobulin. All these are factors associated with a higher risk of symptomatic UTI in the follow-up, as previously stated. Although treatment of AB was an independent risk factor of developing a symptomatic UTI within six months, the presence of other confounding factors not considered in the present analysis could not be ruled out.

Some randomized trials assessing the impact of treatment AB on kidney recipients have already been reported, with results against treating, within small samples [4,27]. Some others are in the process of being concluded or published and might clarify this issue (NCT01871753 and NCT02113774) [27].

## 5. Conclusions

In summary, the high rate of resistant UTIs in kidney recipients and the lack of clinical benefits of treating AB in the present study support the recommendation of stopping screening and treating AB, while waiting for robust incoming assay results. The risk factors for developing a symptomatic UTI observed in this study might help define a subpopulation that could benefit from specific strategies, such as close follow-up, antimicrobial prophylaxis, or self-antibiotic initiation once symptoms are present.

## Figures and Tables

**Table 1:** Baseline, clinical and microbiological features of kidney recipients with bacteriuria.

Variable	All Cases *n* = 197	Asymptomatic Bacteriuria *n* = 175	Cystitis *n* = 22	*p* Value
Time from transplant to inclusion (years; median, IQR)	3.76 (0.78–10.3)	3.85 (0.77–9.92)	2.35 (0.63–11.4)	0.48
Diabetes mellitus- *n* (%)	46 (23.4)	42 (24.0)	4 (18.2)	0.79
Transplant indication- *n* (%)				0.97
Tubulointerstitial	40 (20.3)	35 (20.0)	5 (22.7)	-
Glomerulonephritis	40 (20.3)	36 (20.6)	4 (18.2)	-
Polycystic kidney disease	36 (18.3)	32 (18.3)	4 (18.2)	-
Diabetic nephropathy	11 (5.6)	9 (5.1)	2 (9.1)	-
Hypertension/renovascular	16 (8.1)	15 (8.6)	1 (4.5)	-
Tumoral	4 (2.0)	4 (2.3)	0 (0)	-
Etiology uncertain/unknown	49 (24.9)	43 (24.6)	6 (27.3)	-
Charlson index (median, IQR)	3 (2-5)	3(2-5)	4(2-5)	-
Induction drug- *n* (%)				
None	99 (50.3)	87 (49.7)	12 (54.5)	-
Basiliximab	56 (28.4)	49 (28.0)	7 (31.8)	-
Daclizumab	11 (5.6)	10 (5.7)	1 (4.5)	-
Thymoglobulin	24 (12.2)	23 (13.1)	1 (4.5)	-
Current immunosuppression- *n* (%)				
MMF	142 (72.1)	2.0)	16 (72.7)	-
Prednisone	180 (91.4)	161 (92.0)	19 (86.4)	-
Tacrolimus	174 (88.3)	155 (88.6)	19 (86.4)	-
mTOR inhibitors	10 (5.1)	9 (5.1)	1 (4.5)	-
Cyclosporine	12 (6.1)	10 (5.7)	2 (9.1)	-
Urinary instrumentation- *n* (%)	40 (20.3)	35 (20.0)	5 (22.7)	0.84
Double J stent	34 (17.3)	29 (16.6)	5 (22.7)	-
Urethral catheter	3 (1.5)	3 (1.7)	0 (0)	-
Nephrostomy	3 (1.5)	3 (1.7)	0 (0)	-
Length of instrumentation (days, median, IQR)	0 (0-26)	0 (0–26)	0 (0–43.5)	0.52
Cotrimoxazole prophylaxis- *n* (%)	32 (16.2)	28 (16%)	4 (18.1)	-
Etiology- *n* (%)				
*Escherichia coli*	89 (45.2)	79 (45.1)	10 (45.5)	0.93
*E. coli* ESBL-producers	5 (2.5)	5 (2.9)	0 (0)	0.55
*Klebsiella pneumoniae*	30 (15.2)	28 (16.0)	1 (4.5)	0.15
*K. pneumoniae* ESBL-producers	5 (2.5)	4 (2.3)	1 (4.5)	0.54
*Enterococcus faecalis*	23 (11.6)	20 (11.4)	3 (13.6)	0.76
*Pseudomonas aeruginosa*	13 (6.6)	11 (6.3)	2 (9.1)	0.62
*Klebsiella oxytoca*	8 (4.0)	6 (3.4)	2 (9.1)	0.27
*Proteus mirabilis*	7 (3.6)	6 (3.4)	1(4.5)	0.75
*Morganella morganii*	4 (2.0)	4 (2.3)	0 (0)	0.62
*Enterobacter aerogenes*	4 (2.0)	3 (1.7)	1(4.5)	0.44
*Enterobacter cloacae*	3 (1.5)	2 (1.4)	1 (<1)	0.33
Treatment- *n* (%)	75 (38.1)	54 (30.9)	21 (95.5)	
Ciprofloxacin	22 (11.2)	16 (9.1)	6 (27.3)	0.01
Fosfomycin	29 (14.7)	19 (10.9)	10 (45.5)	<0.01
Amoxicillin-clavulanate	16 (8.1)	12 (6.9)	4 (18.2)	0.07
Cephalosporins	5 (2.5)	4 (2.3)	1 (4.5)	0.53
Cotrimoxazole	3 (1.5)	3 (1.7)	0 (0)	0.54
Antibiotic resistance- *n* (%)				
Ciprofloxacin	55 (27.9)	45 (25.7)	10 (45.5)	0.16
Fosfomycin	19 (9.6)	17 (9.7)	2 (9)	0.94
Amoxicillin-clavulanate	38 (19.2)	31 (17.7)	7 (31.8)	0.12
Cephalosporins	21 (10.6)	16 (9.1)	5 (22.7)	0.08
Cotrimoxazole	60 (30.4)	53 (30.5)	7 (31.8)	0.73

MMF: Mycophenolate, mTOR: Mammalian target of rapamycin, ESBL-producers: extended spectrum beta-lactamases-producers. IQR: Interquartile range.

**Table 2:** Microbiological and clinical outcomes of the total events, asymptomatic bacteriuria and cystitis.

Variables	Bacteriuria *n* =197	AB *n* =175	Cystitis *n* =22	*p* Value
One month follow up outcome Microbiological- *n* (%)				
Cure	111 (56.3)	99 (56.7)	12 (54.5)	0.51
Persistence	40 (20.3)	35 (20.0)	5 (22.7)	0.75
Relapse	11 (5.5)	11 (6.3)	0 (0)	0.26
Re-infection	14 (7.1)	10 (5.7)	4 (18.2)	0.07
Without follow up data	21 (10.6)	20 (11.4)	1 (4.5)	0.35
Clinical- *n* (%)				
Asymptomatic	191 (96.9%)	170 (97.1%)	21 (95.4%)	0.5
Cystitis	4 (2.0)	3 (2)	1 (4.5)	0.6
APN	2 (1.0)	2 (1)	0 (0)	0.6
Six months follow up outcome Microbiological- *n* (%)				
Cure	53 (26.9)	48 (27.4)	5 (22.7)	0.45
Persistence	34 (17.2)	31 (17.7)	3 (13.6)	0.68
Relapse	27 (13.7)	24 (13.7)	3 (13.6)	0.96
Re-infection	37 (18.7)	31 (17.7)	6 (27.2)	0.29
Without follow up data	58 (29.4)	49 (28.0)	9 (40.9)	0.3
Clinical- *n* (%)				
Asymptomatic	181 (91.8)	163 (93.1%)	19 (86.3)	0.22
Cystitis	8 (4.0)	6 (3.4)	1 (4.5)	0.26
APN	8 (4.0)	6 (3.4)	2 (9)	0.26
Graft outcome- *n* (%)				
Graft dysfunction	10 (5.1)	8 (4.6)	2 (9.1)	0.29
Graft rejection	4 (2.0)	4 (2.3)	0 (0)	0.17
Graft loss	1 (0.5)	1 (0.6)	0 (0)	0.17

AB: Asymptomatic bacteriuria, APN: Acute pyelonephritis.

**Table 3:** Characteristics of treated and untreated patients with asymptomatic bacteriuria.

Variables	Treated AB *n* = 54	Untreated AB *n* = 121	OR (95%CI)	*p* Value
Previous creatininemia (mg/dL, median, IQR)	1.78 (0.82–2.75)	1.54 (0.86–2.22)	0.08 (0.048–0.212)	0.07
Creatininemia at the time of inclusion (mg/dL)	1.77 (0.98–2.57)	1.57 (0.88–2.25)	0.02 (0.126–0.157)	0.08
Time since transplant < 6 months	16 (30.2)	20 (16.7)	2.16 (1.013–4.614)	0.04
One month follow up outcome Microbiological- *n* (%)				
Cure	24 (44.4)	75 (61.9)	0.49 (0.25–0.94)	<0.01
Persistence	10 (18.5)	25 (20.6)	0.9 (0.51–1.6)	0.7
Relapse	7 (12.9)	4 (3.3)	2.2 (1.3–3.69)	0.04
Re-infection	7 (12.9)	3 (2.4)	2.4 (1.5–3.9)	<0.01
Without follow up data	6 (11.1)	14 (11.6)	0.97 (0.48–1.9)	0.95
Clinical- *n* (%)				
Symptomatic UTI	4 (7.4)	1 (0.8)		
Cystitis	2 (3.7)	1 (0.8)	2.2 (0.96–5.1)	0.25
APN	2 (3.7)	2 (3.7)	3.3 (2.65–4.2)	0.09
Six months follow up outcome Microbiological- *n* (%)				
Cure	13 (24.1)	37 (30.6)	0.8 (0.4–1.3)	0.3
Persistence	12 (22.2)	25 (20.6)	1.06 (0.6–1.8)	0.8
Relapse	12 (22.2)	14 (11.6)	1.6 (1.01–2.7)	0.06
Re-infection	14 (25.9)	23 (19)	1.3 (0.80–2.12)	0.11
Without follow up data	13 (24.1)	36 (29.8)	0.8 (0.48–1.38)	0.45
Clinical- *n* (%)				
Cystitis	2 (3.7)	3 (2.5)	1.3 (0.44–3.92)	0.66
APN	4 (7.4)	1 (0.8)	2.8 (1.8–4.3)	0.03
Graft outcome- *n* (%)				
Graft rejection	1 (1.8)	3 (2.5)	0.8 (0.14–4.5)	0.8
Graft dysfunction	2 (3.7)	6 (4.9)	0.7 (0.14–3.8)	0.7
Graft loss	0 (0.0)	1 (0.8)	-	0.7

AB: Asymptomatic bacteriuria, APN: Acute pyelonephritis. IQR: Interquartile range.

**Table 4:** Risk factors of symptomatic UTI during the 6 months of follow-up in patients with asymptomatic bacteriuria.

Variables	Symptomatic UTI (*n* = 15)	No Symptomatic UTI (*n* = 182)	Crude OR (95% CI)	*p* Value	Adjusted OR (95% CI)
Urinary pH (median, IQR)	6.7 (6.2–7.2)	6.4 (5.9–6.9)	0.11 (0.01–0.2)	0.06	-
Previous creatininemia (mg/dl, median, IQR)	2.07 (0.87–3.27)	1.55 (0.84–2.27)	0.06 (0.01–0.12)	0.02	-
Time after transplant < 1 year (median, IQR)	9 (60)	49 (27.22)	4.01 (1.4–11.9)	0.01	5.7 (1.4–22.2)
Recurrent UTI previous transplant- *n* (%)	4(26.7)	28 (15.3)	-	0.3	-
Urinary reflux- *n* (%)	0 (0.0)	13 (7.1)	-	0.3	-
MMF doses (median, IQR)	700 (435–1045)	750 (329–1268)	-	0.6	-
Induction treatment- *n* (%)	11 (73.3)	80 (43.9)	3.2 (1.1–9.7)	0.01	-
No drug	4 (26.7)	95 (52.2)	0.36 (0.12-1)	0.05	-
Basiliximab	4 (26.7)	52 (28.7)	-	1.0	-
Daclizumab	1 (6.6)	10 (5.5)	-	1.0	-
Thymoglobulin	6 (40)	18 (9.9)	4.6 (1.8–11.8)	<0.01	8 (1.9–34.2)
Previous APN post-transplant- *n* (%)	11 (73.3)	60 (32.9)	4.8 (1.6–14.7)	<0.01	12 (2.7–53.5)
Developing UTI 2 months after transplant- *n* (%)	13 (86.7)	93 (51.1)	4.6 (1.1–19.8)	0.03	-
Previous rejection- *n* (%)	2 (13.3)	12 (6.6)	-	0.49	-
Urinary instrumentation- *n* (%)	5 (33.3)	35 (19.2)	-	0.36	-
Obstructive uropathy post-transplant- *n* (%)	0 (0.0)	10 (5.5)	-	0.59	-
Nosocomial acquisition of the AB- *n* (%)	11 (73.3)	167 (91.7)	0.24 (0.06–0.99)	0.04	-
Antibiotic therapy of the AB- *n* (%)	11 (73.3)	64 (35.2)	4.7 (1.5–13.5)	0.02	5 (1.2–20.6)
Microbiological cure at 1 month- *n* (%)	4 (26.6)	107 (58.8)	0.2 (0.09–0.854)	0.01	-

UTI: Urinary tract infection, MMF: Mycophenolate, APN: Acute pyelonephritis, AB: Asymptomatic bacteriuria.
